# Computational modeling of fatigue crack propagation in butt welded joints subjected to axial load

**DOI:** 10.1371/journal.pone.0218973

**Published:** 2019-06-27

**Authors:** Oscar Araque, Nelson Arzola, Omar Varón

**Affiliations:** 1 Department of Mechanical Engineering, Universidad de Ibagué, Ibagué, Colombia; 2 Research Group in Multidisciplinary Optimal Design, Departamento de Ingeniería Mecánica y Mecatrónica, Universidad Nacional de Colombia, Bogota, Colombia; University of Vigo, SPAIN

## Abstract

This article addresses the study of crack behavior elicited on axial fatigue in specimens joined by butt weld made of steel ASTM A36 by using fracture mechanics and simulation software of finite elements (Ansys APDL, Franc3D). The computational model was initially to define the geometry model by using CAD software. Specimens with Weld Reinforcement of 2 mm and 3mm were simulated. Subsequently, the type of element for the mesh, the information inclusion concerning material mechanical properties and load conditions were selected. By using Franc3D software, the crack propagation phenomenon is analyzed, and its growth parameters have been established. In this way, it is possible to calculate the magnitude of stress intensity factor (SIF) along the crack front. It is concluded that the stress located in the weld toe is maximized proportionately to the size of the weld reinforcement due to the concentration effect of geometric stress. In addition, it is observed that the propagation rate obtained from Paris law has a similar behavior for the studied weld reinforcements; the latter as there were short cracks.

## Introduction

The integrity of welded joints has always been a concern in the engineering field. Most of these joints are found in elements subjected to cyclical loads, thus the incidence of flaws such as cracks are inevitable. These cracks emerge because of different factors among which changes in microstructures in steels are highlighted, inclusions that can generate stress concentration or simply operation conditions. This phenomenon behavior is studied by Fracture Mechanics, which is defined as the study of crack propagation in an elastic material through determination of critical conditions (for instance, magnitude and type of load and defect size) for which their growth is produced [1].

Regarding the analysis of fracture mechanics of welded joints, several authors have researched the issue in the last years through the selection of a combination of factors, for example, material, geometry of the model, and load conditions. However, most have chosen the use of computational tools and the finite elements method (FEM). For instance, Berrios [2] has found a prominent correspondence among experimental trajectories of crack propagation in high-strength steel and trajectories of propagation obtained by using ANSYS APDL software.

However, there are some international standards that must be considered in the design of metal structures (where there are welded joints). Standards such as BS 7910 [3] that establish the failure criteria, as, fracture, fatigue, creep and other types of failure (corrosion and buckling), as well as to replace traditional standards in welded joints. This allows it for greater discontinuities in areas subjected to small stress which saves resources without compromising the safety of the structure. From another perspective, Hobbacher [4] establishes a basis for the design and analysis of welded joints whose stress can be considered fluctuating to avoid fatigue failures. The evaluation procedures determine the information related to the action and fatigue resistance. However, these methods depend, in turn, on available data of the welded joint and the forces involved. On the other hand, Newman [5] has proposed a series of empirical equations of stress intensity factors for elliptical, semi-elliptical cracks, among others, which are embedded in a finite body subjected to axial loads, They are especially useful when crack propagation rates are to be analyzed and the calculation of fracture toughness in the types of faults mentioned above. Likewise, Bowness [6] has developed a mathematical model for the determination of the magnifying factors (Mk) in the welding foot for semi-elliptical cracks in welded joints in T. The author concluded that the equations described should not be applied to the calculation of K deep in the tip of the crack due to a singularity fault of r.

On the other hand, Yusuke [7] has researched the effects of cracks configuration and residual stress characteristic of weld in Weibull stress for high-strength steel concluding that residual stresses decrease the embedded crack’s fragile fracture limit before global deformation reaches the yield stress. Other researches [8], have studied the influence of weld bead geometry in a lifespan of a jointing compound of two welded steel plates A36 by an electrode E6013. It was concluded that a higher reinforcement of the weld bead has a consequence a reduction of up to 42% of fatigue life. Moreover, Citarella [9] has done an approximation to the numerical growth cracks prediction for fatigue under certain load spectrum through a FEM-DBEM approach and has inferred a satisfactory agreement between numerical and experimental crack propagation when using the formula of two parameters. As well as the existence of flexibility and efficiency in the methodology adopted because FEM and DBEM were complementary in this study. Similarly, Fumiaki [10] has examined the mechanism of crack branching and the effect of surface roughness in Nickel steels through experimentation and the use of the Finite Element Method. The author has concluded that stress triaxiality tends to increase through crack propagation and critical condition for crack branching is when the main stress exceeds 1500 MPa. Likewise, Younise [11] has studied based on a numerical model, the onset and development of a ductile fracture in welded joints of high-strength steel, finding that strength in the onset and crack propagation is significantly affected by welding heterogeneity.

Within the same research field, Lewandowski [12] has analyzed crack growth behavior in ferrite-perlite structures subjected to cyclic bending and has determined that, in all cases, life in fatigue of welded specimens is lower than those fully solid due to dissimilar mechanical properties of characteristic zones of welded joints (base, bead, and ZAT). In addition, Zerbst [13] has researched fracture mechanics implementation in the establishment of fatigue strength in welded joints whose cracks were originated in the weld toe. He has found that the mechanical properties and S355NL and S960QL steels in the heat-affected zones differ in the correspondence between simulations and experiments. This fundamentally occurs because of higher-strength steel weld toe deformation is still elastic for applied higher stress.

A new finite elements method with interface elements has been developed by Serizawa [14] with the purpose of examining microstructural fracture behavior and where the anisotropy of grain was modeled by ordinary finite elements. It has been found that anisotropic mechanical properties of grain boundary are a prevailing factor in the fracture process. Following this line of work, Salcedo-Mora [15] has exposed a meshfree microstructural elements method to analyze the microstructure effect in quasi-fragile properties within numerical simulations of damage, improving computational accuracy and cost in engineering applications; the researcher has demonstrated a method for released-energy standardization as for calculations made from the Finite Element Method on thick mesh. It allows the conducting of simulations for Finite Elements for deformations development on thick mesh without losing accuracy in the result. Similarly, Szávai [16] has proposed a thermal-metallurgical-mechanical tridimensional model of finite elements with the aim of researching the residual stress microstructure and distribution of a welded joint among dissimilar metals in a pressurized vessel. This author has found there is an acceptable agreement between predicted and measured data. Besides, both the numerical model and the experiment show that strengthening by deformation is the cause of final residual stress. Comparably, Guo [17] has developed a numerical model of tridimensional fracture by using the combined finite-discrete element method with the purpose of providing a base for engineering applications. It has been demonstrated that accuracy in tridimensional fracture modelling depends on the size of the element around crack fronts. One of the most used models for crack propagation in mechanical lineal fracture is Paris law on which researchers as Ciavarella [18], Ancona [19], Carrascal [20], Kirane [21] [22], Rajabipour [23] and Toribio [24] have carried out their studies from different perspectives.

The use of fracture analysis software, for example, Franc3D has gained popularity among researchers in the last decade. Yang [25], for instance, has formulated an algorithm based on the mechanic of lineal elastic fracture, for cracks growth simulation for fatigue under non-proportional loads. He found out that growth cycles measured based on stress intensity factors (SIF) satisfactorily correspond to experimental information for specimens subjected to slightly lower loads. Likewise, Xiao [26] has undertaken a numerical analysis for cracks of a semispherical cavity surface and has concluded that geometry of the cavity surface is determining for SIF. With regard to a more specific context, Chin [27] has researched how a crack is propagated in a vessel made from a titanium and aluminum alloy (very common in aerospace industry) and has found that Mode I (SIF’s) is found below toughness to the material fracture. Therefore, the vessel could withstand certain type of cracks. Equally, Liu [28] applied mechanics of lineal fracture and FEM tools for the analysis of a reactor pressure vessel to calculate stress intensity factors in a given embedded crack front. The research results suggest that the crack growth rate nodes in the reactor internal surface is faster than nodes in the central area of the crack front.

As stated above, research in Fracture Mechanics field is based on the evaluation of specific phenomena enabled by certain fractal mechanical parameters such us the stress intensity factor. The aim of this research is the calculation of crack propagation in welded joints subjected to cyclic axial load; as main characteristics the use of a none-standardized geometry is node, commonly used in structural joints and the inclusion in the simulation model of two welding leg sizes.

## Materials and methods

A modified version of the standard specimen for fatigue pursuant to ASTM E8/E8M – 09 regulations has been used in this study. It is an idealized version of steel plates ASTM A36 joined through a weld bead done by electrode E6013. Considering that the welding objective is to reach certain uniformity at a metallurgical level, the assumption of a single material for the specimen turns reasonable. The experimental design was oriented to determine the set of conditions within finite elements software that might simulate, approximately, a uniaxial fatigue testing. The foregoing means to define all properties of final simulations into a factorial arrangement was applied. It consisted in two factors (weld bead reinforcement of 2 and 3mm) and three levels (weld reinforcement bending, meshing, failure position) in each factor.

The methodology used in this research consisted in the creation of CAD modeling software, as for the purpose of stress-deformation analysis along the specimen before crack growth, incorporation and crack growth. Finally, the construction of Paris curve from crack stable propagation, from stress intensity factors detected by the software (FRANC3D).

**Fig 1:**
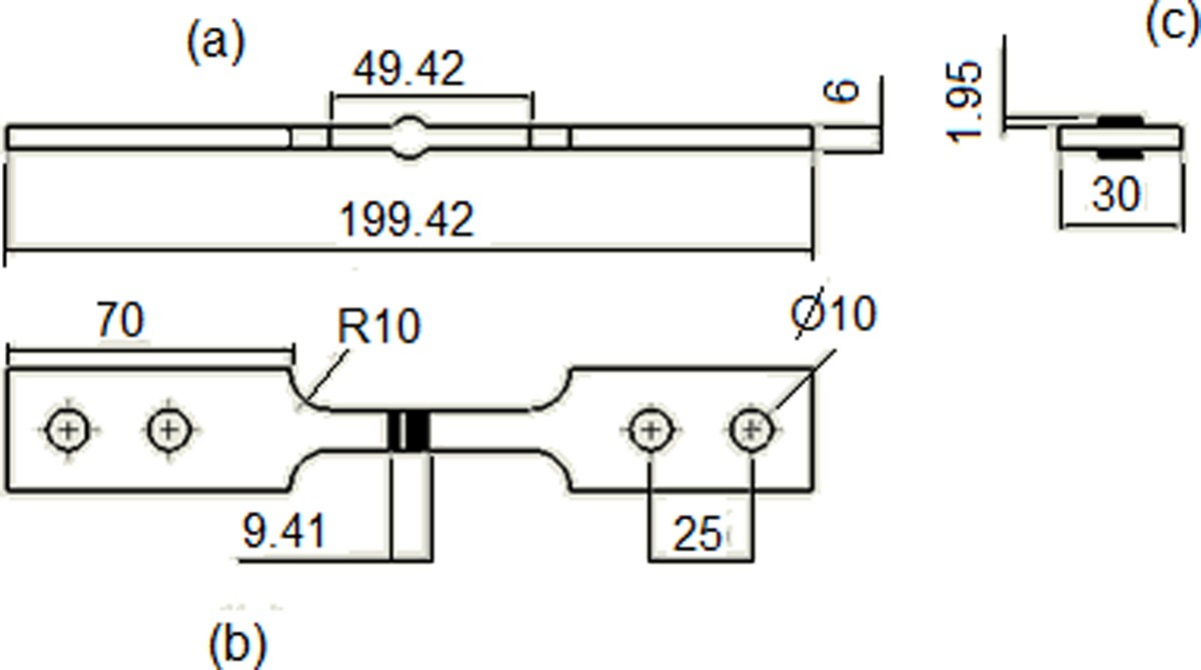
Views: front (b), top (a) and side (c).

Dimensions (in millimeters) of the specimen are shown in Fig 1. Two different specimens were used, the most significant geometric variation is in the weld bead toe, the geometry corresponding to a weld reinforcement of 2mm is the one shown in the figure.

**Fig 2:**
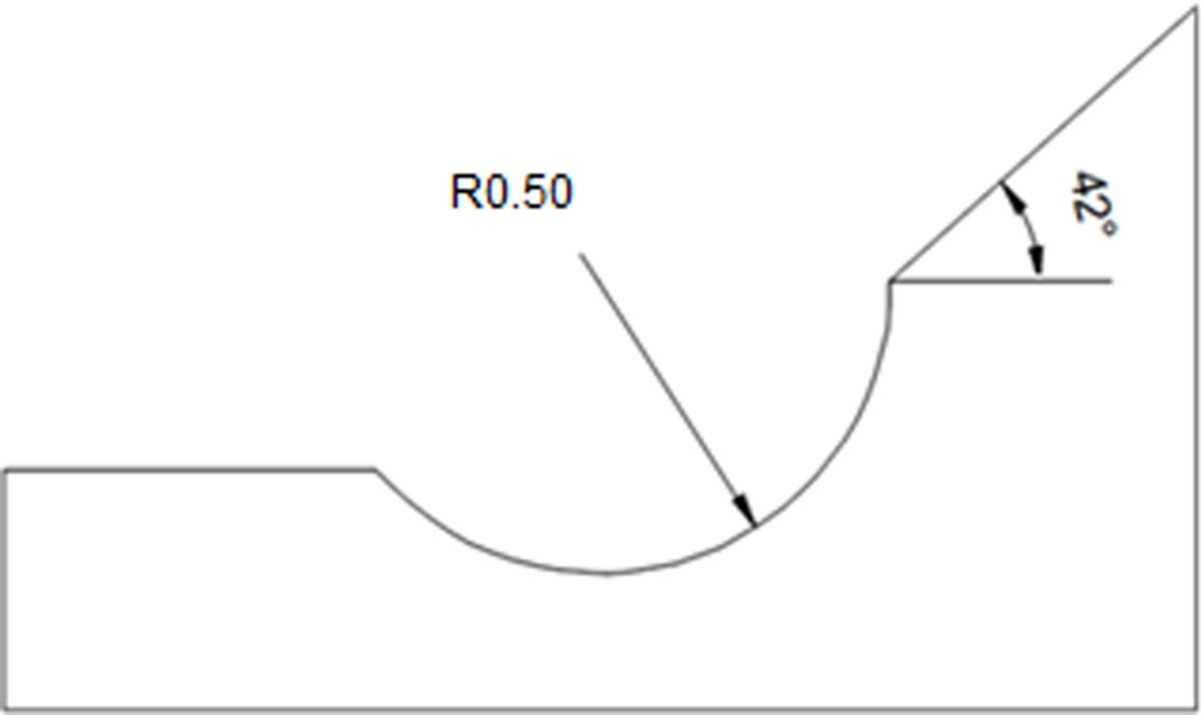
Local weld toe geometry.

As regards geometry the weld of toe, identical for the two specimens used, it was characterized as shown in Fig 2. This weld of toe was graphically represented as a grinding treatment. According to the authors [29] and [30] the value of the toe radius from which life begins to be optimized in fatigue is, on average, from 0.5 mm.

To calculate crack growth rate, it is imperative to determinate the stress intensity factors (SIFs) for load modes I and II using the following equations [31]:

$${\text{K}}_{\text{I}}=\frac{\mu }{\text{k}+1}▪\sqrt{\frac{2\pi }{\text{L}}}▪\left\{4\left({\text{v}}_{\text{b}}-{\text{v}}_{\text{d}}\right)+\left({\text{v}}_{\text{e}}-{\text{v}}_{\text{c}}\right)\right\}$$ (1)

$${\text{K}}_{\text{II}}=\frac{\mu }{\text{k}+1}▪\sqrt{\frac{2\pi }{\text{L}}}▪\left\{4\left({\text{u}}_{\text{b}}-{\text{u}}_{\text{d}}\right)+\left({\text{u}}_{\text{e}}-{\text{u}}_{\text{c}}\right)\right\}$$ (2)

with:

$$\mu =\frac{\text{E}}{2\left(1+\nu \right)}$$ (3)

$$\text{k}=\{\begin{array}{c} 3-4\nu \;\;\;\;\;\;\left(\text{plane}\;\text{strain}\right)\\ \frac{3-\nu }{1+\nu }\;\;\;\;\left(\text{plane}\;\text{stress}\right) \end{array}$$ (4)

where:․

K_I_, K_II_: Stress intensity factors for load modes I and II, respectively (MPa m^1/2^).
E: Material Elasticity Module (MPa).
v: Poisson’s Coefficient (dimensionless).
L: Characteristic Length of the singular element (mm).
(u_i_; v_i_): Displacements in x and y, respectively of the i node of the singular element (mm).

**Fig 3:**
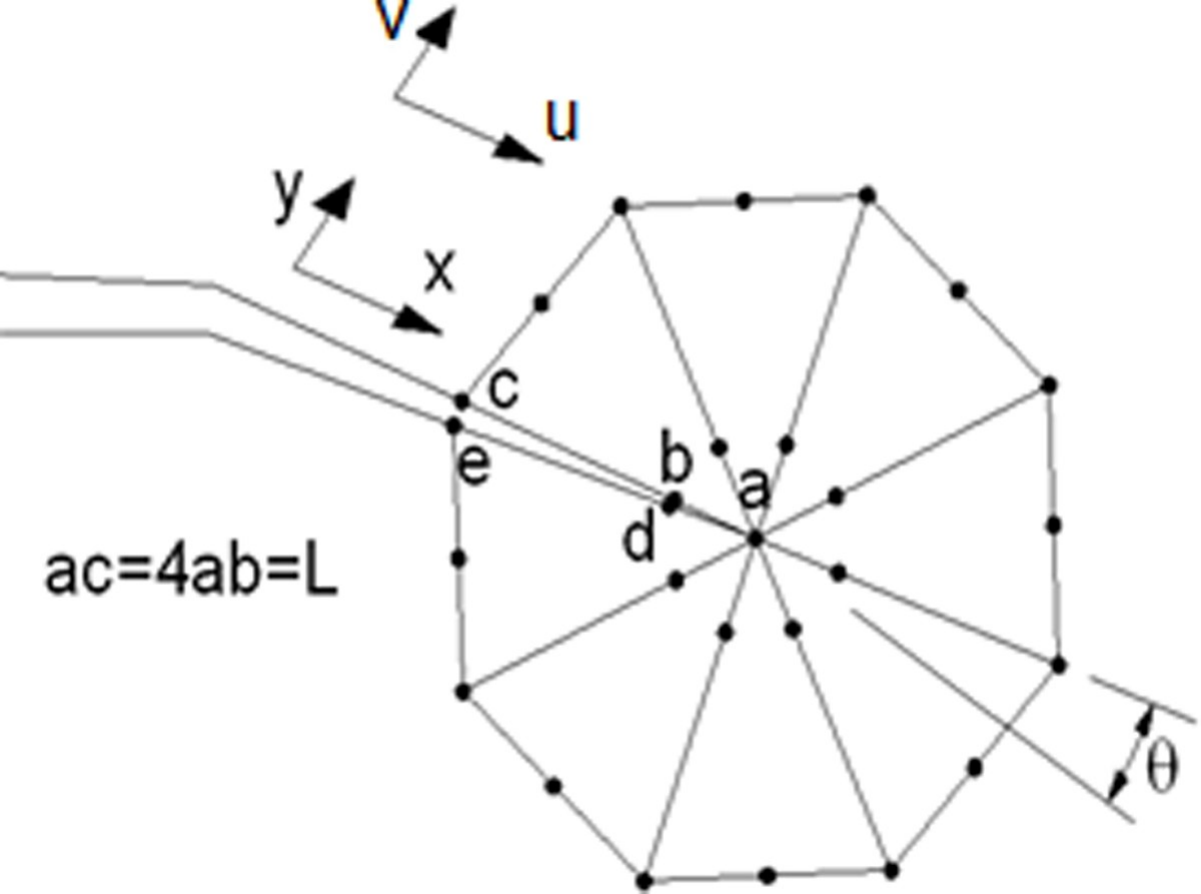
Arrangement of control nodes on the crack vortex.

Fig 3 shows the singular elements, node arrangement, and displacements used in calculating the *stress intensity factors*.

To obtain accurate results, meshing of the component is done by observing some rules. For example, the crack front must be meshed with small singular concentric elements and which do not vary in size as the crack extends. The rest of the component is meshed with quadrangular elements that provide good precision.

Another approach in the determination of KI is found in Anderson analytical model [32] for semi-elliptical cracks in bodies subjected to traction. Equations that describe this phenomenon are set out below:

$$K_{I}=\sigma_{m}\sqrt{\frac{\pi a}{Q}}F\left(\frac{a}{t},\frac{a}{c},\frac{c}{W},\emptyset \right)$$ (5)

Where:

$$Q=1+1.464{\left(\frac{c}{a}\right)}^{1.65}$$ (6)

$$F=\left[M_{1}+M_{2}{\left(\frac{a}{t}\right)}^{2}+M_{3}{\left(\frac{a}{t}\right)}^{4}\right]f_{\varphi }f_{w}g$$ (7)

$$M_{1}=\;\sqrt{\frac{c}{a}}\left[1+0.04\left(\frac{c}{a}\right)\right]$$ (8)

$$M_{2}=\;0.2{\left(\frac{c}{a}\right)}^{4}$$ (9)

$$M_{3}=\;-0.11{\left(\frac{c}{a}\right)}^{4}$$ (10)

$$g=1+\left[0.1+0.35\left(\frac{c}{a}\right){\left(\frac{a}{t}\right)}^{2}\right]{\left(1-sin\emptyset \right)}^{2}$$ (11)

$$f_{\varphi }={\left[{\left(\frac{c}{a}\right)}^{2}sin^{2}\emptyset +cos^{2}\emptyset \right]}^{\frac{1}{4}}$$ (12)

$$f_{w}={\left[sec\left(\frac{\pi c}{2W}\sqrt{\frac{a}{t}}\right)\right]}^{\frac{1}{2}}$$ (13)

**Fig 4:**
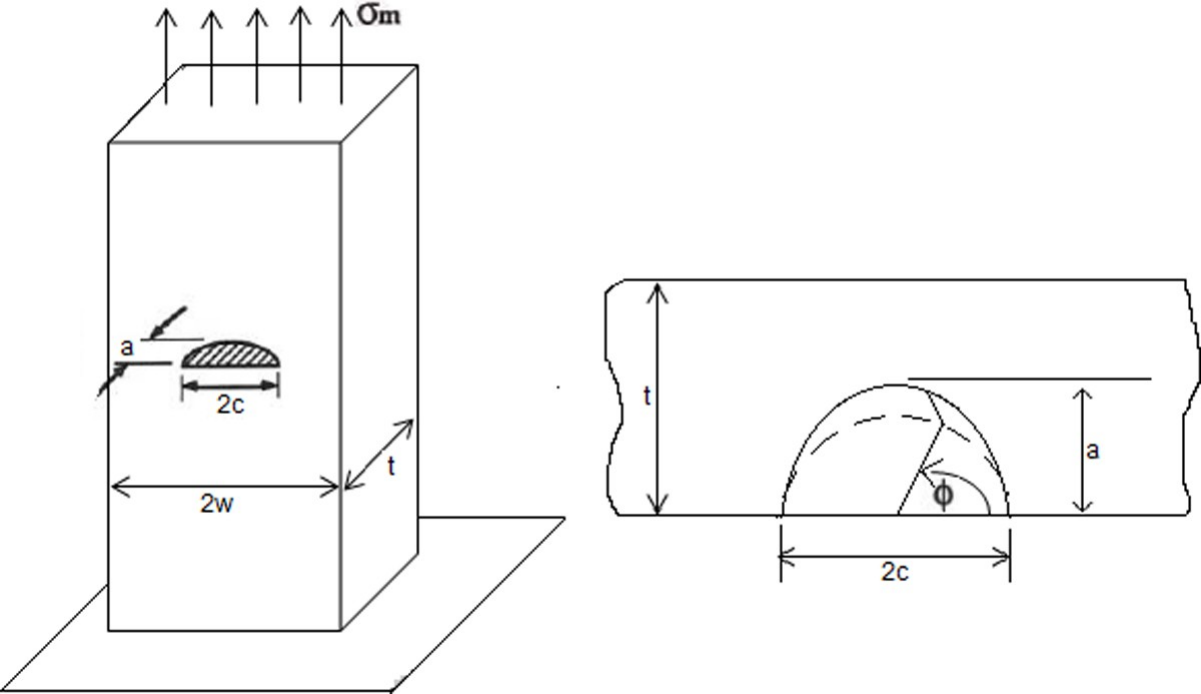
Element subjected to an axial stress. Adapted from [2].

Fig 4 shows the corresponding model to a semi-elliptical crack subjected to an axial load.

To estimate the rate of crack propagation for a given initial geometry, the Paris-Erdogan law is used [33].

$$\frac{da}{dN}=C\Delta K_{I}^{m}$$ (14)

Where $\frac{da}{dN}$ is the crack growth rate and it is expressed in mm/cycle; ΔKI is the rise of SIF and it is expressed in $MPa\sqrt{m}$; C and m are the constant of Paris Law, which depend on the material and in this case they have been considered as $6.89\;*10{\;}^{-12}\;MPa\sqrt{m}$ y 3, respectively.

### Numerical processing

**Fig 5:**
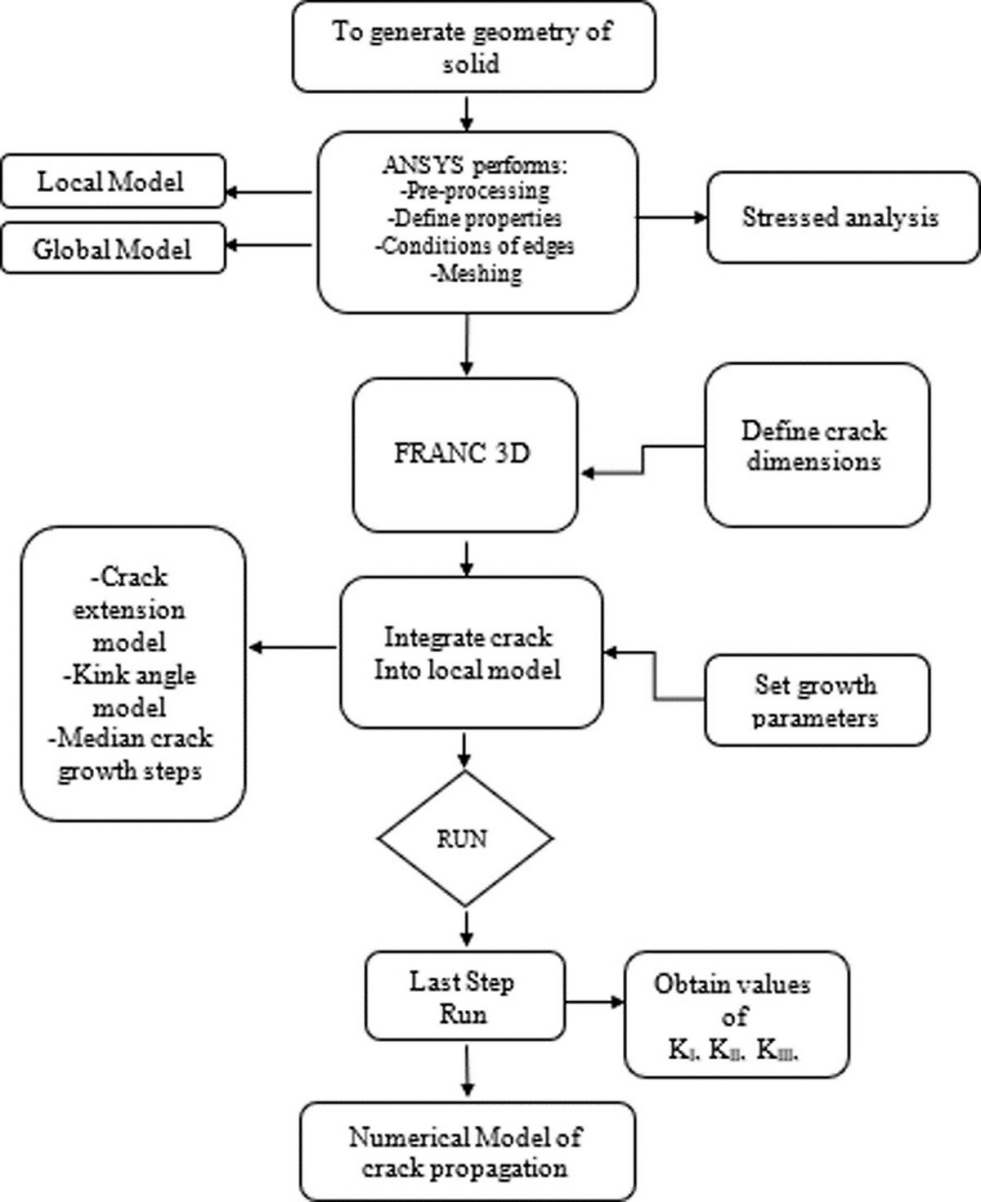
Modeling approach for crack propagation.

Fig 5 Methodology is expressed in a comprehensive manner for the computing analysis conducted to each specimen. It should be noted that most of the processing capacity used comes from ANSYS APDL, because the 3D FRANC software relies on the latter for the tasks of calculation and information processing.

**Fig 6:**
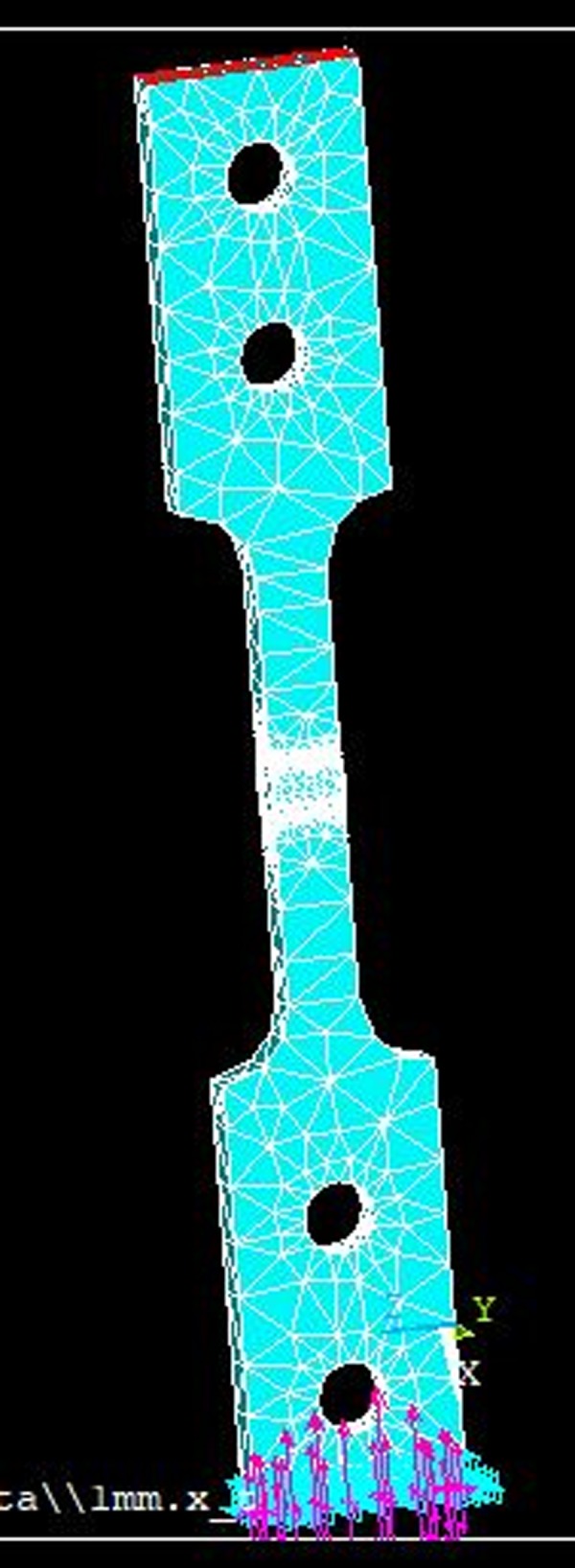
Loads and constraints in the specimen.

Then, once CAD (Solidworks 2016) is built in software, the specimen was directly imported to the software ANSYS APDL. As shown in Fig 6; the model is subjected to a tensile stress of 68.9 MPa at the top (red region) and, in the opposite side, a movement constraint has been imposed in all directions (purple region) emulating its tensile test.

For the development of the simulation Quadratic Tetrahedral Elements were used, these elements are adapted to complex geometries besides having more degrees of freedom; They also allow a greater degree of refinement than is necessary to facilitate the crack growth Regarding the properties of the steel, an elastic and isotropic linear material with the mechanical properties detailed in Table 1 is used.

**Table 1:** Mechanical properties of steel ASTM A36 *[32]*.

Mechanical Properties	Magnitude
Tensile Strength Yield	250 MPa
Tensile Strength Ultimate	400–550 MPa
Modulus of Elasticity	200 GPa
Poisson's Ratio	0.26
Shear Modulus	79.3 GPa

#### Crack growth

**Fig 7:**
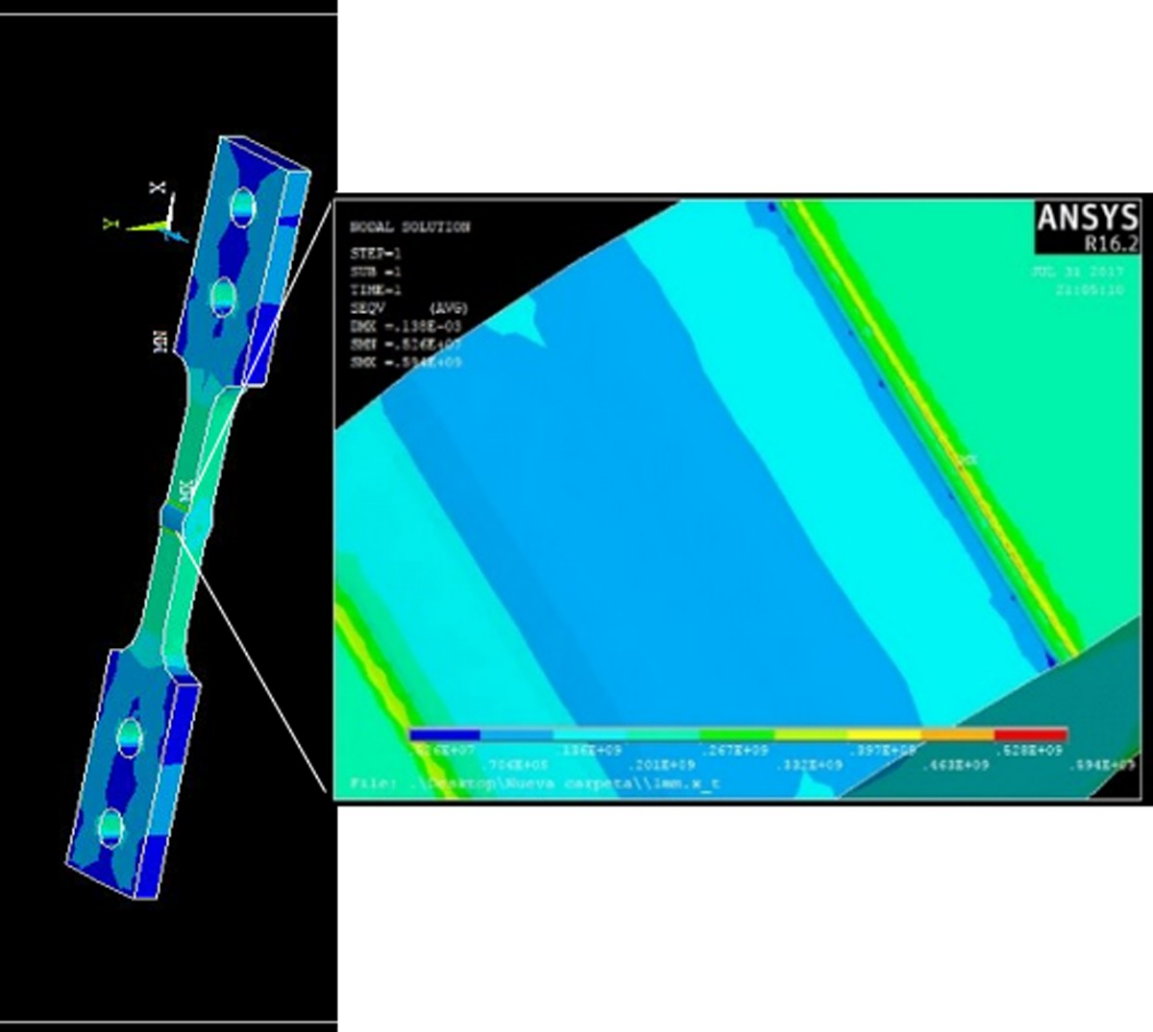
Stress distribution (Von Mises).

The failure location in the model previously described was not done in a random manner. This means that to find the suitable region to implement the crack, it was first necessary to make a stress analysis as shown in Fig 7.

**Fig 8:**
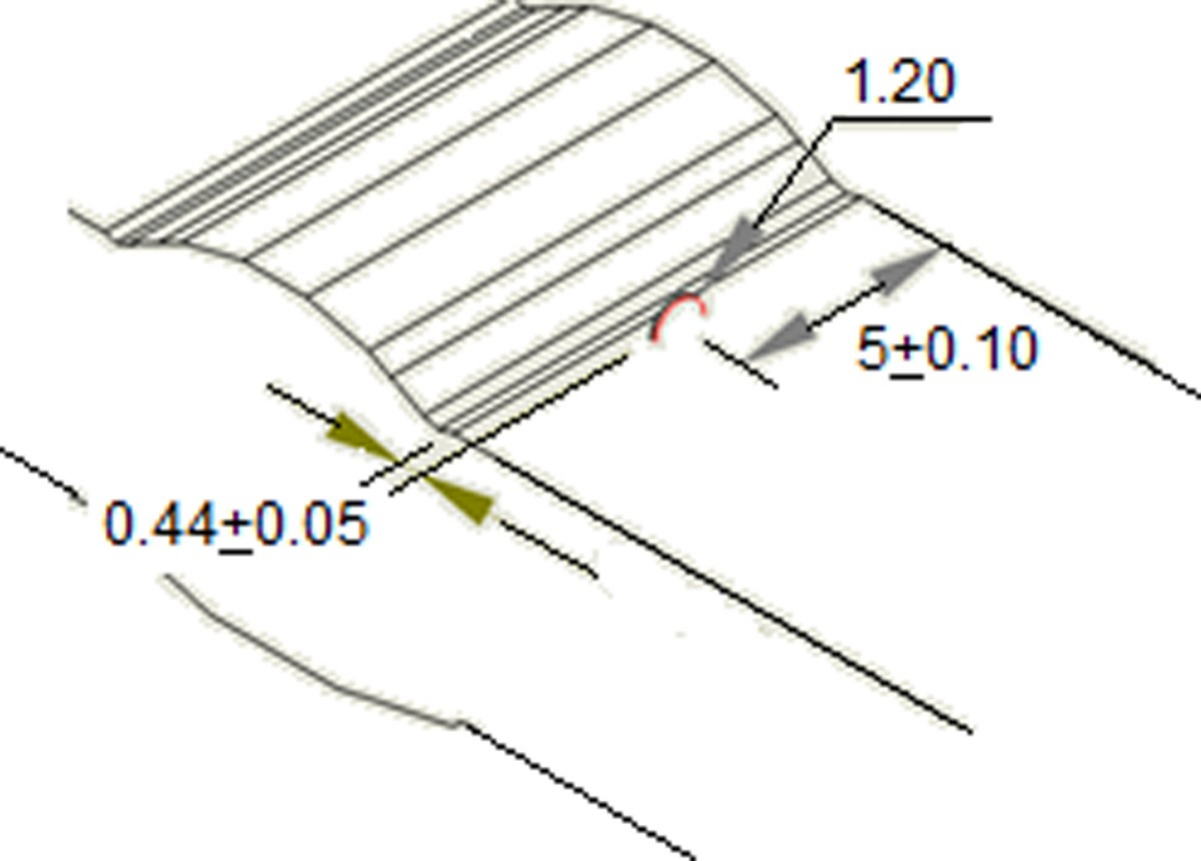
Location of the flaw in welding.

From the computational simulation it is found that the largest stress concentration is in the region near the weld bead (yellow region), with a maximum stress located at the start of the crack propagation of 549 MPa. Once the area where the crack might be located (As shown in Fig 8), it was written, using ANSYS, a file type CDB, hence the format is compatible with FRANC3D.

**Fig 9:**
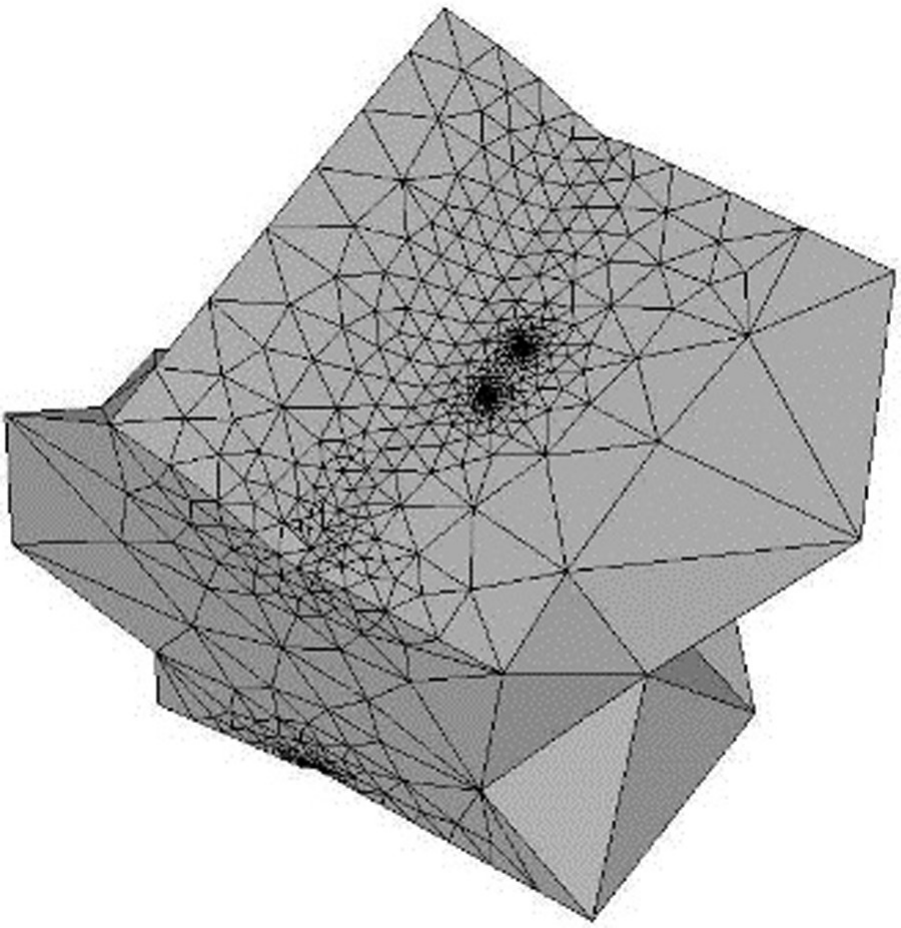
Crack inserted in the model (FRANC3D).

After having imported the model of this software, the crack is inserted as shown in Fig 9 and the simulation of the propagation of this is initiated, having set growth parameters (growth model, failure size, number of steps, etc.).

The reason why Fig 9 does not show the total model observable in Fig 7 is because the division in local and global models was fundamental. This means that to achieve the optimization of processing capacity of ANSYS APDL, the crucial part of the model was divided where the crack was located, decreasing this way, the presence of needless elements. Once the crack had grown until the limit of the number of elements supported by the used version of ANSYS, then information was obtained from SIF’s (Stress Intensity Factors) and respective Paris crack propagation Curves were built. The former procedure was developed for specimens whose reinforcement in the weld bead corresponded to 2mm and 3mm.

## Discussion and results

### Verification of numerical models

The most suitable numerical model of finite elements for the computational processing is selected, by referencing the Stress Intensity Factor (KI), three numerical models were formulated and the relative error was determined for each of these models, from the theoretical value of KI estimated according to [32]. Then, bearing in mind that Table 2. can be observed that the balance point between time in CPU and the percentage of error was found close to the Numerical Model 1, thus it was the type of mesh selected for simulations.

**Table 2:** Computational Cost Vs. Relative Error.

	Nodes	Elements	CPU Time (min)	KI (MPa sqrt(m))	% Error
Analytical Model of Anderson				4.410	
Numerical Model 1	68426	41758	45	4.338	1.62
Numerical Model 2	63124	38294	35	4.099	7.04
Numerical Model 3	49892	30260	25	4.127	6.40

### Crack propagation behaviour

**Fig 10:**
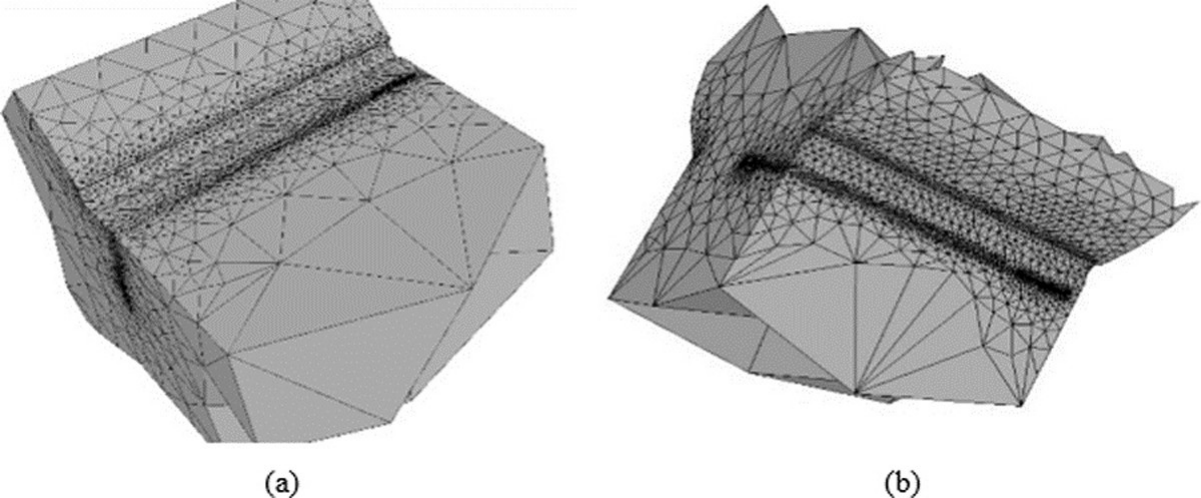
Growing crack. (a) Weld reinforcement 2 mm. (b) Weld reinforcement 3 mm.

In this section analysis results of propagation carried out under loads exclusively of tensile for two sizes of weld reinforcement are presented. The growth rate for each case corresponds to the following characteristics: the initial crack width has 1.2 mm (measure in the semi-major axis of the ellipse) located approximately in the half of the weld toe length and the configuration corresponds to a linear increase depth of 0.12 mm applied to a total of 50 steps of length. For the specimens whose weld bead had a reinforcement of 2mm and 3mm; the crack propagation was just as it is shown in Fig 10.

**Fig 11:**
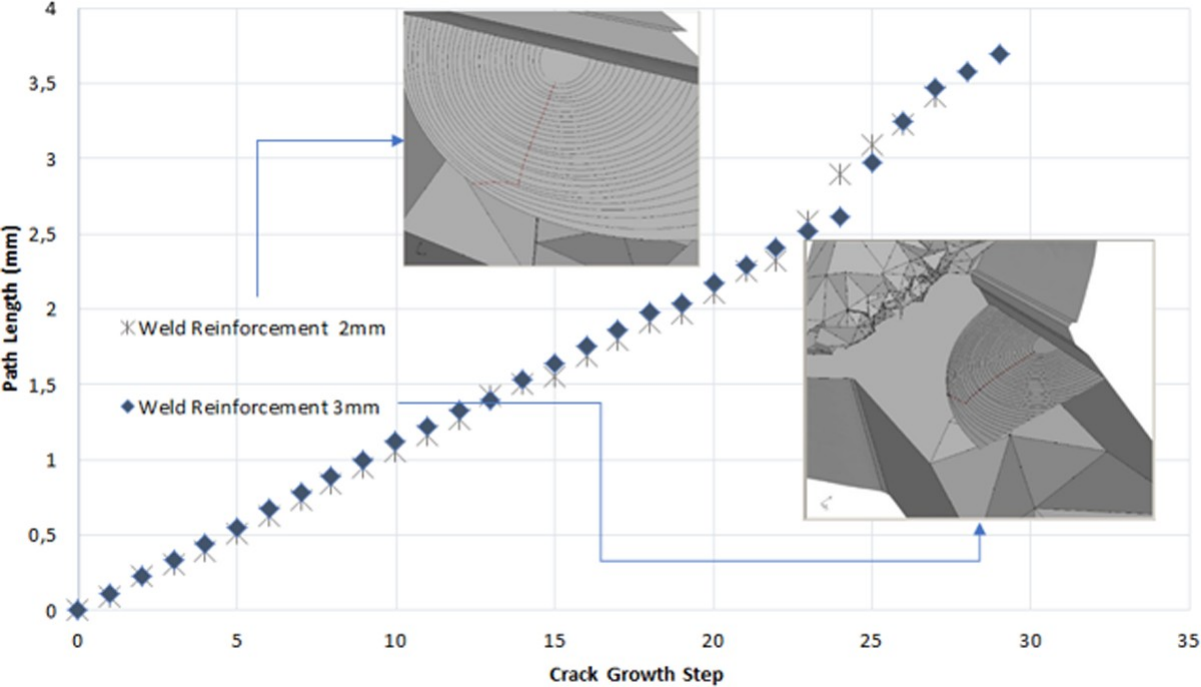
Crack growth in both weld reinforcement.

In Fig 11 shown the way the crack size increases for each growth step. The change of propagation paths (red line) observable in the graph are explained due to a change in Fig 10.

It is possible to appreciate how grooves are pulling away from each other as the crack is growing, anticipating a major propagation rate and an imminent break.

**Fig 12:**
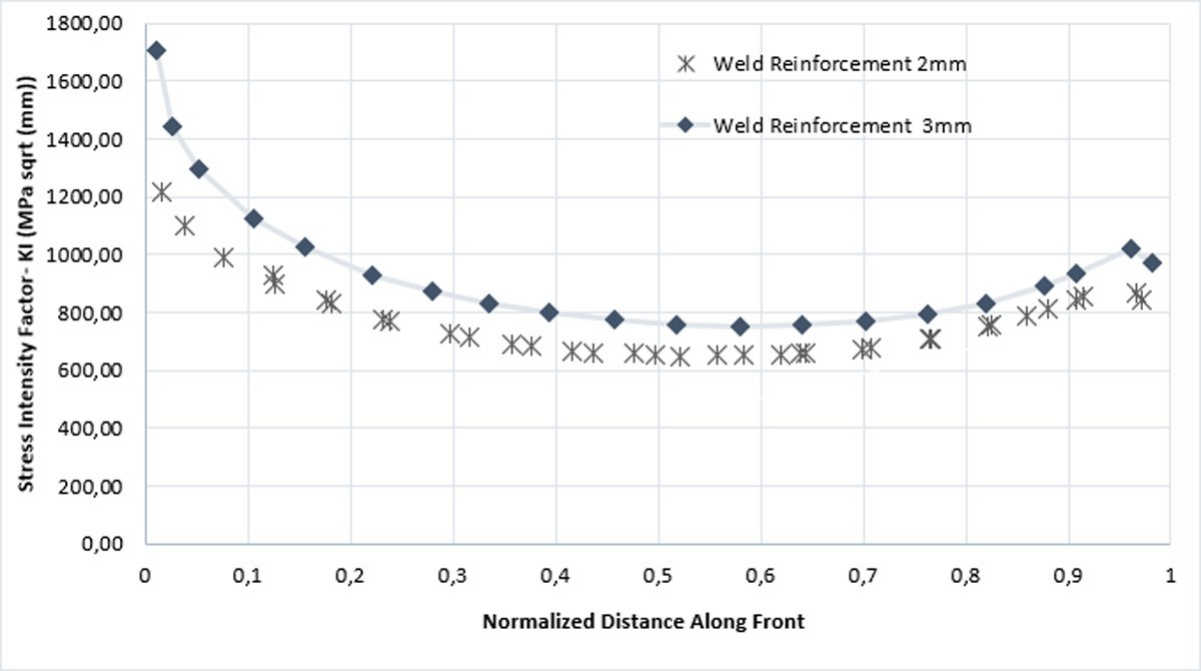
Stress intensity factor KI in function of the crack front length.

Consecutively, in Fig 12 values of *k*_*1*_ are shown throughout crack front for each step. The maximum points were found at the crack front corresponding to a size of 3.4 mm and 3.7 mm, for the weld reinforcement of 2 mm and 3mm respectively.

On the other hand, the former graphs show that there was always a change of surface in the propagation for specimens of 2 mm and 3 mm of weld reinforcement, the magnitude of the stress intensity factor for this crack front becomes significantly higher when the crack front distances more from the initial surface of propagation [34].

**Fig 13:**
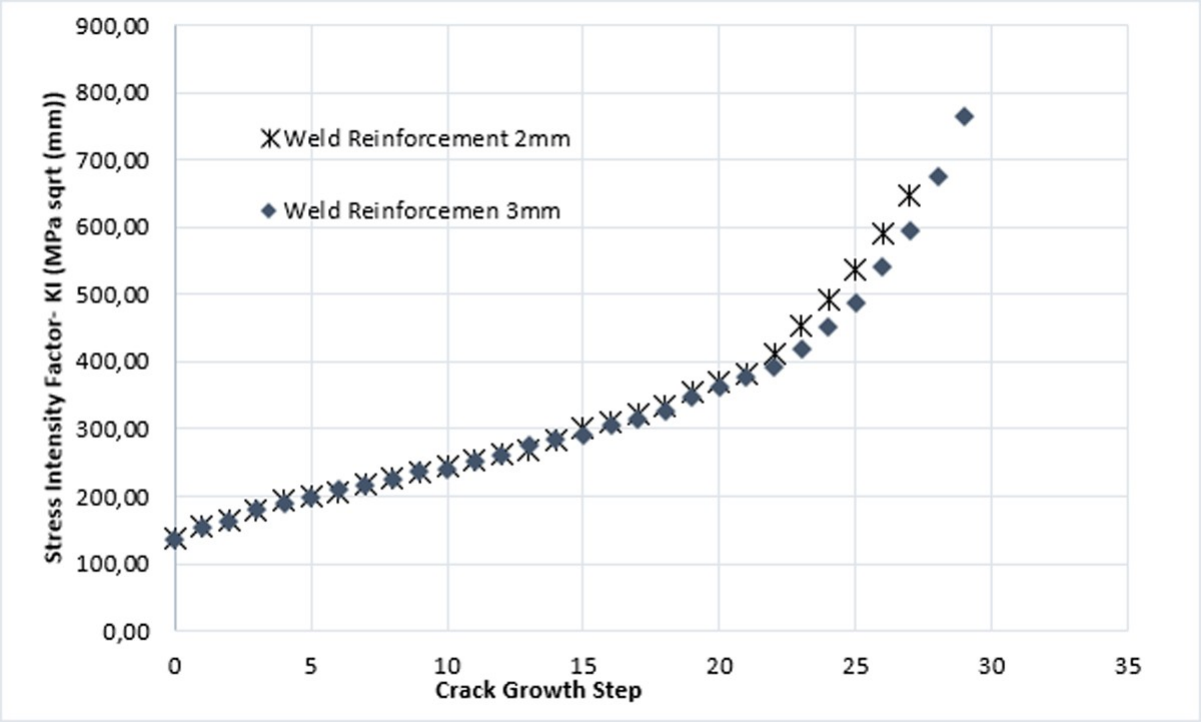
Stress intensity factor KI in function of growth.

In Fig 13 crack growth step is illustrated and the rise of stress intensity factor for each step. It is shown how the distribution of stress is practically the same in the propagation until step number 22 where a gradual break of KI values starts for both geometric configurations.

**Fig 14:**
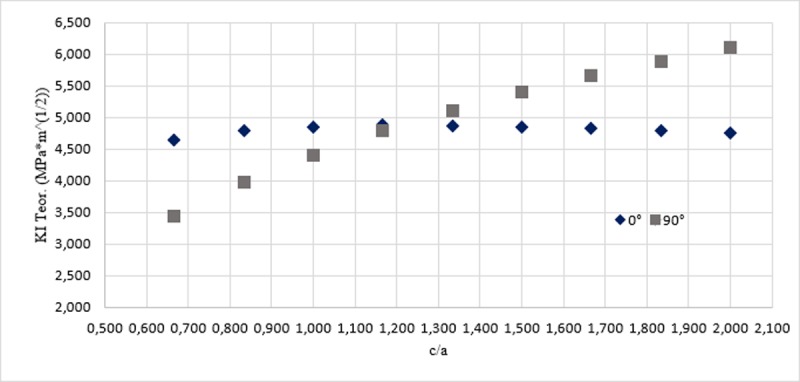
Variation of KI in function of the geometric parameter c/a.

**Fig 15:**
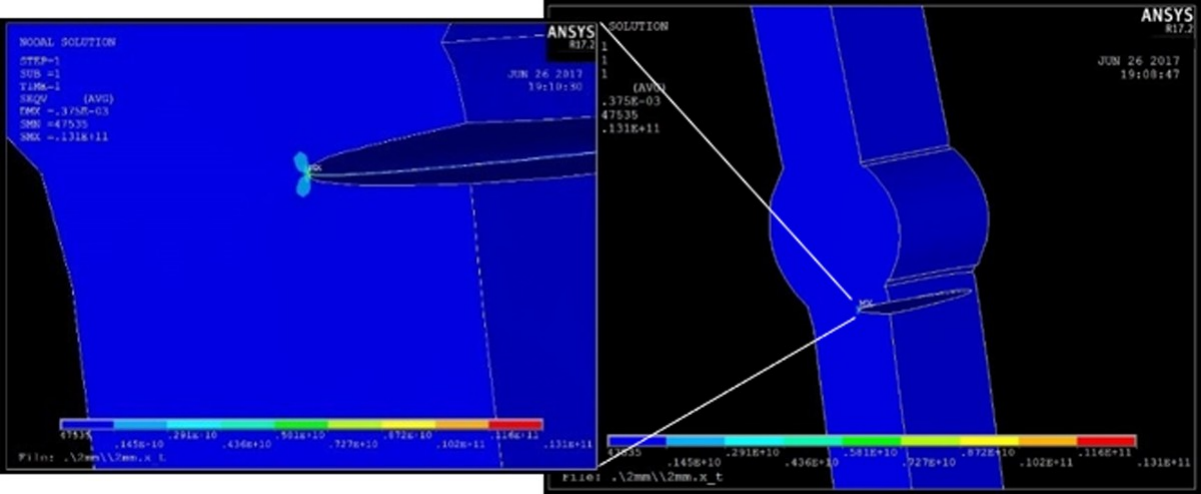
Stress in the crack front for reinforcement of 2 mm.

**Fig 16:**
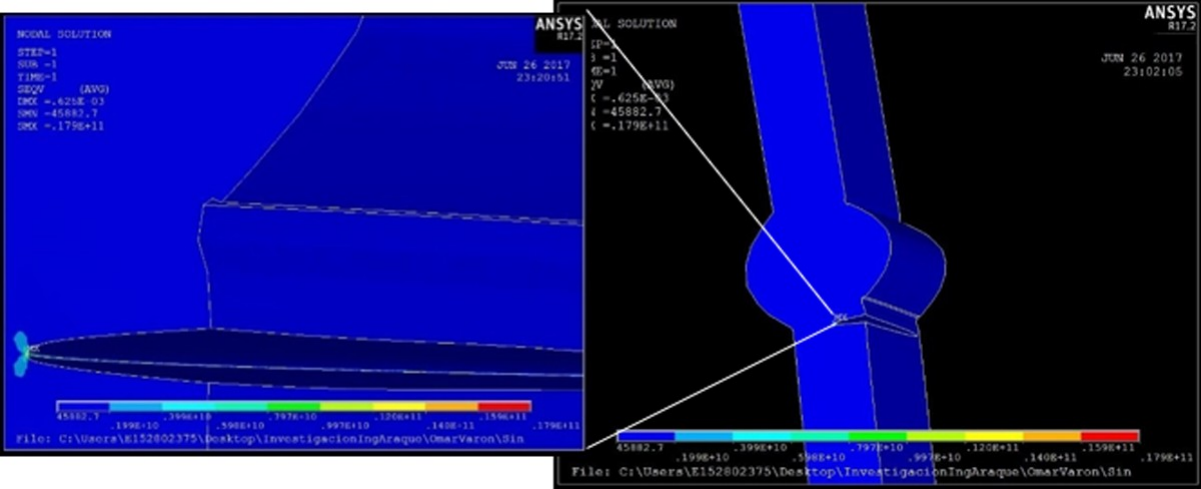
Stress in the crack front for reinforcement of 3 mm.

According to the set out above, it in inferred that the largest magnitudes of KI are found in the ends of each front, fact that was contrasted in two ways: the first by analyzing a singularity in Anderson’s analytical model illustrated in Fig 14, and the second, by the stress map in the area affected by the failure, as shown in Figs 15 and 16, for the reinforcement of 2 mm and 3 mm respectively. In Fig 14, the geometric parameter *c/a* is associated, which is a relation between the major axis of the ellipse and the distance embedded in the specimen, that, in this case, is the half of the whole of the minor axis and the angle φ for the stress intensity factor. The Stress Intensity Factor (KI) estimate was carried out by using the Eq (5). The foregoing is consistent with what was stated by the researcher Takahashi in [35], indicates that, the maximum stress intensity factor occurring between the vertex points where the nominal values are calculated.

As revealed in Fig 14, when the relation c/a is 1, the ellipse has a void eccentricity, thus, geometrically, is a circumference; when comparing KI for each angle it is observed clearly that for 0°, the value KI is higher, compared to if it were measured in the deepest point of the crack (90°). However, this ceases to apply when rising the focal distance of the ellipse by leaving invariant the minor axis; therefore, from c/a equal 1,2 the higher magnitude of KI will be found in an angular position of 90°.

**Fig 17:**
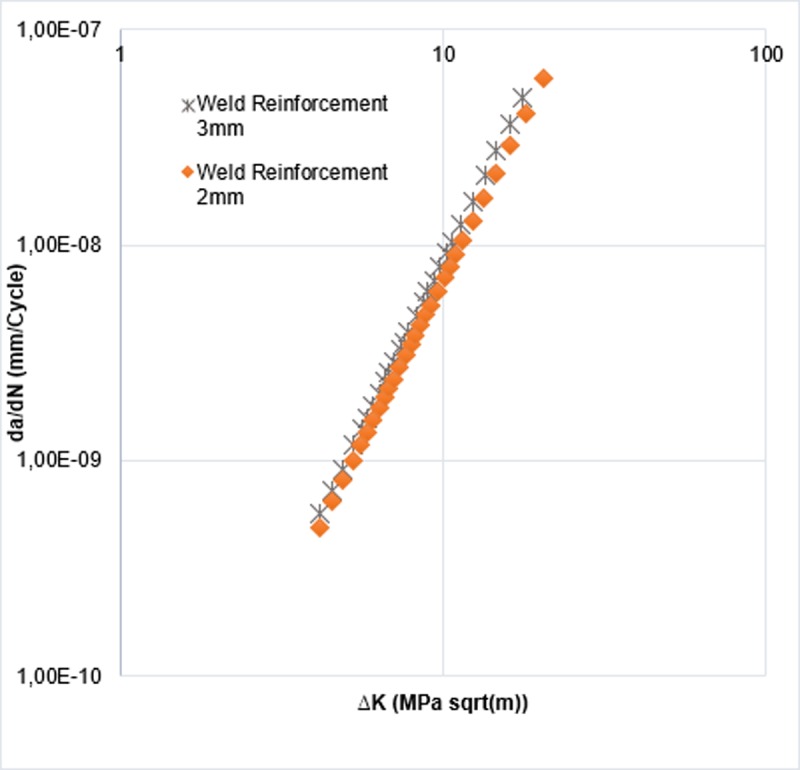
Crack propagation rate for the two reinforcement sizes studied.

About the magnitude of the local stress, the computational analysis confirms that the reinforcement of the bead is proportional to the concentration of efforts in the crack tip, by observing that there were no large plastic deformations, for that reason the material elastic range was enough to describe the stress behavior in the specimen. It was possible to establish the growth rate for each specimen as shown in Fig 17 by using the Paris model.

Paris law allows to estimate the number of produced cycles to reach the preset progress in each iteration, in which considering front shape variation of an iteration to the following determines the crack propagation rate for the studied models. It is observed that weld reinforcement had no significant effect in the crack growth speed, for the two types of weld reinforcement considered in this study. The above is related to the short crack size of the subjects of study models. When analyzing Fig 11, discrepancies are observed in the growth of the crack (a) in the last steps of the simulation that correspond to the change of the propagation surface (see Fig 10). Therefore, Regarding the initial size and the growth rate of the crack remained constant for both cases, it is possible to affirm that these are small numerical errors of the software.

## Conclusions

The Stress Intensity Factor (SIF) has been determined throughout a semi-elliptical crack front. To that end, a numerical model has been used by applying the Finite Element Method (FEM) using ANSYS and FRANC3D software, which allows the variation of SIF on the crack front. In the study two weld reinforcements length have been considered, observing that most of the magnitudes of KI are found on the edges of each front, fact related to the presence of frame geometric eccentricities. Predicting crack propagation by using the Paris law shows no significant effect on the weld size, in the studied range, regarding steady growth speed of the crack. It is possible to confirm that the 3mm weld reinforcement results in an increase in the SIF on average of 0.5–2% higher than the 2mm weld reinforcement, which implies an increase in the crack growth rate greater than 6.1%. Furthermore, the little difference between the Paris curves is due to the effect of the stress concentration factor near of the weld toe.

## Supporting information

S1 Table Crack growth step for both weld reinforcement. a) 2mm b) 3mm.
(PDF)

S2 Table Stress intensity factor KI in function of the Normalized distance along front. (PDF)

S3 Table Stress intensity factor KI in function of crack growth step. (PDF)

S4 Table Paris Model parameters for crack propagation rate a) weld reinforcement 2mm, b) weld reinforcement 3mm.
(PDF)

## References

[1] Universidad Nacional de la Plata [Internet]. Departamento de Aeronáutica; c2019 [cited 2019 Jan 12]. Available from: http://www.aero.ing.unlp.edu.ar/catedras/archivos/Mecanica%20de%20Fractura%202010rev01.pdf

[2] Berrios D, Franco R. Análisis Experimental y Numérico de la Trayectoria de Propagación de Fisuras por Fatiga Utilizando XFEM. Información Tecnológica (SciELO). 2018; 29(5): 19–34.

[3] British Standard, BS 7910: Guide on Methods for Assessing the Acceptability of Flaws in Metallic Structures.,BSI, London, Standard 2013.

[4] Hobbacher Adolf, Recommendations for fatigue design of welded joints and components. New York: Springer, 2015.

[5] Raju IS Newman JC, An empirical stress intensity factor equation for the surface crack, Engineering Fracture Mechanics, vol. 15, pp. 185–192, 1981.

[6] Lee MMK, Bowness D, "Prediction of weld toe magnification factors for semi-elliptical cracks in T-butt joints,". International Journal of Fatigue, vol. 22, pp. 369–387, 2000.

[7] Seko Y, Imai Y, Mitsuya M. Effects of crack configuration and residual stress on fracture driving force for welded joint with embedded flaw. Procedia. 2016; 2: 1708–1715.

[8] Arzola N, Araque O. Chord profile influence on the fatigue failure of a t-butt weld joint. Ingeniare. 2014; 22 (2): 196–204.

[9] Citarella R, Lepore M, Perrella M. Coupled FEM-DBEM Simulation of 3D Crack Growth under Fatigue Load Spectrum. Procedia. 2016; 2: 2631–26142.

[10] Fumiaki T, Tomoya K, Shuji A. Study on Crack Branching Condition for Brittle Crack Propagation in Steels. Procedia. 2017; 6: 269–275.

[11] Younise B, Rakin M, Gubeljak N. Numerical prediction of ductile fracture resistance of welded joint zones. Procedia. 2016; 2: 753–760.

[12] Lewandowski J, Rozumek D. Cracks growth in S355 steel under cyclic bending with fillet welded joint. Theoretical and Applied Fracture Mechanics. 2016; 86: 342–350.

[13] Zerbst U, Madia M. Fatigue Strength and Life Determination of Weldments based on Fractur Mechanics. Procedia. 2017; 7: 407–414.

[14] Serizawa H, Tomiyama S, Tsuyoshi Hajima. Basic Analysis of Microstructural Fracture Behavior in Structural Materials by Using FEM with Interface Element. Procedia. 2011; 10: 556–561.

[15] Saucedo-Mora L, Marrow J. FEMME: A multi-scale Finite Element Microstructure MEshfree fracture model for quasi-brittle materials with complex microstructures. Engineering Fracture Mechanics. 2015; 147: 355–372.

[16] Szávai S, Bézi Z. Material Characterization and Numerical Simulation of a Dissimilar Metal Weld. Procedia. 2016; 2: 1023–1030.

[17] Guo L, Xiang J. A numerical investigation of mesh sensitivity for a new three-dimensional fracture model within the combined finite-discrete element method. Engineering Fracture Mechanics. 2016; 151: 70–91.

[18] Ciavarella M, Papangelo A. On the distribution and scatter of fatigue lives obtained by integration of crack growth curves: Does initial crack size distribution matter?. Engineering Fracture Mechanics. 2018; 191: 111–124.

[19] Ancona F, Palumbo D. Automatic procedure for evaluating the Paris Law of martensitic and austenitic stainless steels by means of thermal methods. Engineering Fracture Mechanics. 2016; 163: 206–219.

[20] Carrascal I, Casado J.A., Diego S. Determination of the Paris' law constants by means of infrared thermographic techniques. Polymer Testing. 2014; 40: 39–45.

[21] Kirane K, Bazant Z. Size effect in Paris law and fatigue lifetimes for quasibrittle materials: Modified theory, experiments and micro-modeling. International Journal of Fatigue. 2016; 83(2): 209–220.

[22] Kirane K, Bazant Z. Size effect in Paris law for quasibrittle materials analyzed by the microplane constitutive model M7. Mechanics Research Communications. 2015; 68:60–64.

[23] Rajabipour A, Melchers R. Application of Paris’ law for estimation of hydrogen-assisted fatigue crack growth. International Journal of Fatigue. 2015; 80: 357–363.

[24] Toribio J, Matos J, Gonzales B. Paris Law-Based Approach to Fatigue Crack Growth in Notched Plates under Tension Loading. Procedia. 2017; 5: 1299–1303.

[25] Yang Y, Vormwald M. Fatigue crack growth simulation under cyclic non-proportional mixed. International Journal of Fatigue. 2017; 102: 37–47.

[26] Xiao X, Yan X. A numerical analysis for cracks emanating from a surface semi-spherical cavity in an infinite elastic body by FRANC3D. Engineering Failure Analysis. 2008; 15: 188–192.

[27] Chin P (Rensselaer Polytechnic Institute, Hartford, Connecticut). Stress Analysis, Crack Propagation and Stress Intensity Factor Computation of a Ti-6Al-4V Aerospace Bracket using ANSYS and FRANC3D. Final Report. 2011.

[28] Liu R, Huang M, Peng Y. Analysis for crack growth regularities in the nozzle-cylinder intersection area of Reactor Pressure Vessel. Annals of Nuclear Energy. 2018; 112: 779–793.

[29] Shiozaki T, Yamaguchi N, Tamai Y, Hiramoto J, Kazuhiro O. Effect of weld toe geometry on fatigue life of lap fillet welded ultra-high, International Journal of Fatigue. 2018; 116: 409–420.

[30] Pang H. L. J. Analysis of weld toe radius effects on fatigue weld toe cracks. International journal of pressure vessels and piping. 1994; 58(2): 171–177.

[31] Barsoum R. On the use of isoparametric finite elements in linear fracture mechanics. Numerical Methods in Engineering. 1976; 10(1): 25–37.

[32] Anderson TL. Fracture Mechanics. 3rd ed Florida Taylor & Francis; 2005.

[33] Paris P, Erdogan F. A critical analysis of crack propagation laws. Journal of basic engineering. 1963; 85(4): 528–533.

[34] Araque O, Arzola N, Hernandez E. The Effect of Weld Reinforcement and Post-Welding Cooling Cycles on Fatigue Strength of Butt-Welded Joints under Cyclic Tensile Loading. Materials. 2018; 11(4): 1–19. 10.3390/ma11040594 PMC5951478 29649117

[35] Ritchie I, Karihaloo R, Milne B. Comprehensive structural integrity: Cyclic loading and fatigue, 4th ed: Elsevier, 2003, vol. 4

