# Supplementary figures and images for: Computational modeling of fatigue crack propagation in butt welded joints subjected to axial load
Source: PLoS One. 2019 Jun 27;14(6):e0218973. doi: 10.1371/journal.pone.0218973 (PMC6597091; doi:10.1371/journal.pone.0218973)

**S1 Table.** Crack growth step for both weld reinforcement. a) 2mm b) 3mm

| 2 mm        |      | 3 mm        |      |
|-------------|------|-------------|------|
| Length (mm) | Step | Length (mm) | Step |
| 0           | 0    | 0           | 0    |
| 0,09541     | 1    | 0,10685     | 1    |
| 0,22489     | 2    | 0,23429     | 2    |
| 0,30673     | 3    | 0,33135     | 3    |
| 0,38422     | 4    | 0,44169     | 4    |
| 0,50865     | 5    | 0,55268     | 5    |
| 0,62435     | 6    | 0,66344     | 6    |
| 0,73153     | 7    | 0,77863     | 7    |
| 0,84189     | 8    | 0,88588     | 8    |
| 0,94424     | 9    | 0,99548     | 9    |
| 1,0568      | 10   | 1,12171     | 10   |
| 1,16827     | 11   | 1,21756     | 11   |
| 1,27759     | 12   | 1,3239      | 12   |
| 1,42002     | 13   | 1,39815     | 13   |
| 1,50025     | 14   | 1,5247      | 14   |
| 1,55097     | 15   | 1,63975     | 15   |
| 1,68644     | 16   | 1,75023     | 16   |
| 1,79619     | 17   | 1,86654     | 17   |
| 1,91052     | 18   | 1,98636     | 18   |
| 1,96575     | 19   | 2,03614     | 19   |
| 2,10688     | 20   | 2,16286     | 20   |
| 2,24374     | 21   | 2,28373     | 21   |
| 2,31797     | 22   | 2,4085      | 22   |
| 2,58942     | 23   | 2,51471     | 23   |
| 2,89497     | 24   | 2,61712     | 24   |
| 3,08993     | 25   | 2,97223     | 25   |
| 3,22074     | 26   | 3,23713     | 26   |
| 3,4156      | 27   | 3,47037     | 27   |
|             |      | 3,57408     | 28   |
|             |      | 3,70048     | 29   |

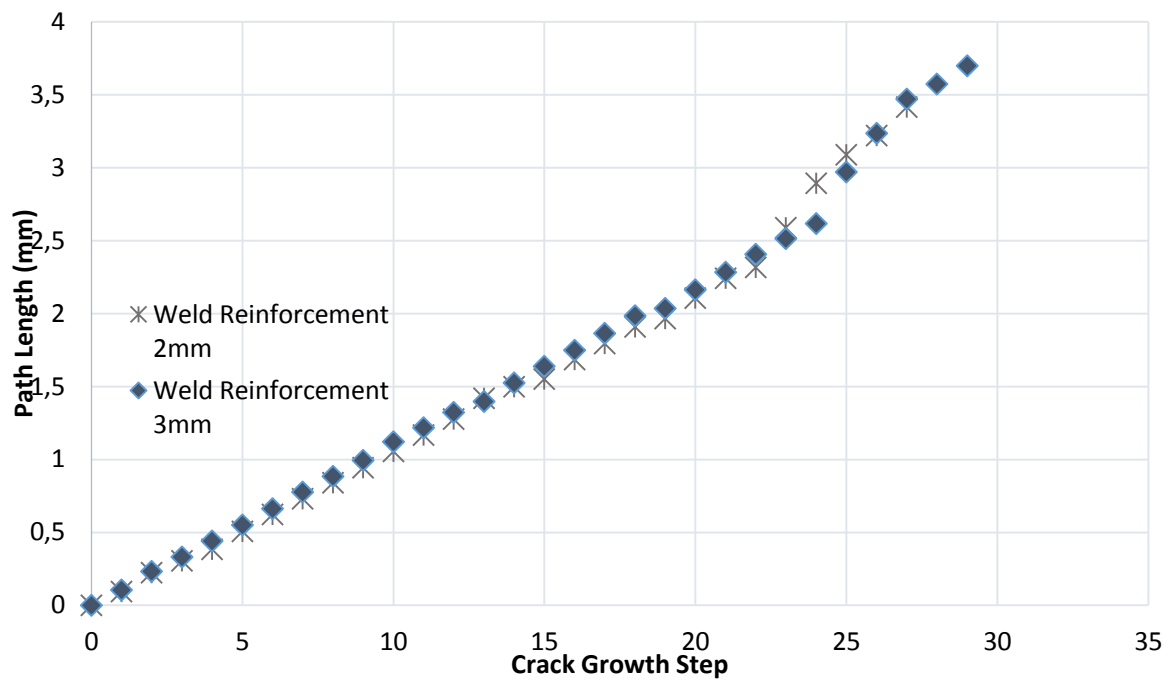

Supplement: S1 Table — a) 2mm b) 3mm. (PDF) [file pone.0218973.s001.pdf]
