# Supplementary material for: Computational modeling of fatigue crack propagation in butt welded joints subjected to axial load
Source: PLoS One. 2019 Jun 27;14(6):e0218973. doi: 10.1371/journal.pone.0218973 (PMC6597091; doi:10.1371/journal.pone.0218973)
Supplement: S2 Table — (PDF) [file pone.0218973.s002.pdf]

S2 Table. Stress Intensity Factor KI in function of the Normalized distance along front.

2mm	
Normalized distance along front	KI (MpaSQRT(m))
0,01556	1216,09
0,03947	1102,40
0,07696	989,41
0,12446	931,58
0,12696	900,24
0,17696	840,15
0,18196	828,86
0,23195	775,36
0,23945	769,41
0,29695	727,77
0,31695	715,88
0,35695	692,10
0,37694	686,15
0,41694	668,32
0,43694	662,37
0,47694	656,43
0,49694	650,48
0,52194	644,54
0,55693	650,48
0,58443	651,24
0,61943	654,37
0,63943	656,65
0,64443	657,44
0,69942	674,26
0,70692	680,21
0,76442	709,93
0,76692	711,23
0,82192	751,58
0,82442	757,52
0,85941	787,25
0,87941	811,03
0,90691	840,75
0,91441	852,64
0,96691	870,51

3mm	
Normalized distance along front	KI (MpaSQRT(m))
0,01034	1704,28
0,02697	1445,45
0,05184	1296,85
0,10675	1122,23
0,15674	1025,12
0,22178	927,88
0,27935	875,89
0,33443	830,35
0,39454	797,72
0,45717	777,95
0,51729	758,19
0,57994	751,33
0,6401	757,40
0,70277	769,89
0,76296	795,31
0,82065	833,67
0,87586	891,41
0,90851	936,38
0,96125	1019,93
0,98134	972,24

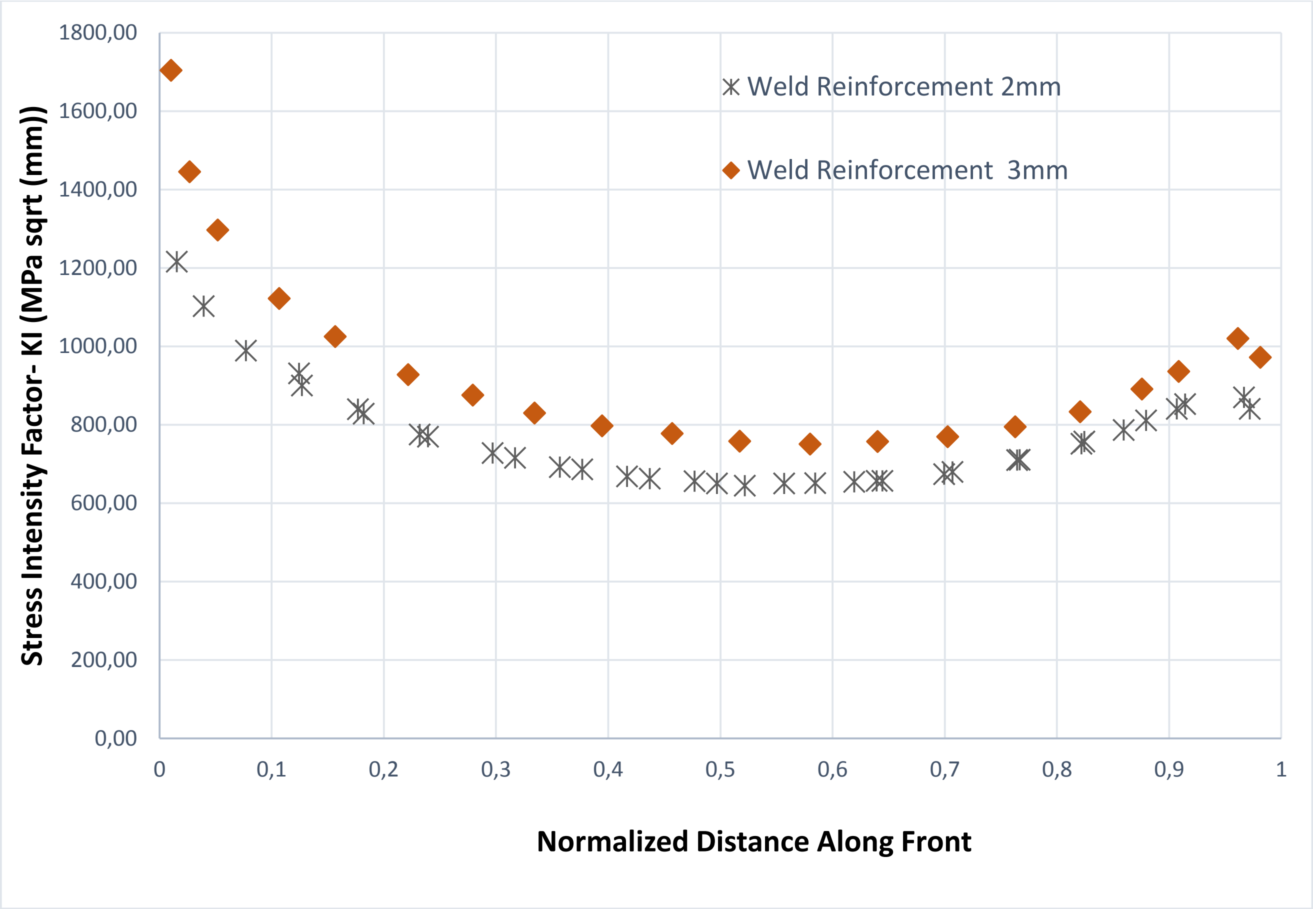

Weld Reinforcement 2mm
Weld Reinforcement 3mm
Stress Intensity Factor- KI (MPa sqrt (mm))
1800,00
1600,00
1400,00
1200,00
1000,00
800,00
600,00
400,00
200,00
0,00
0
0,1
0,2
0,3
0,4
0,5
0,6
0,7
0,8
0,9
1
Normalized Distance Along Front
