# Supplementary material for: Computational modeling of fatigue crack propagation in butt welded joints subjected to axial load
Source: PLoS One. 2019 Jun 27;14(6):e0218973. doi: 10.1371/journal.pone.0218973 (PMC6597091; doi:10.1371/journal.pone.0218973)
Supplement: S3 Table — (PDF) [file pone.0218973.s003.pdf]

**S3 Table.** Stress Intensity Factor KI in function of crack growth step

| 2 mm | | 3 mm | |
|---|---|---|---|
| KI (MPa sqrt(mm)) | Step | KI (MPa sqrt(mm)) | Step |
| 137,47 | 0 | 136,19 | 0 |
| 154,99 | 1 | 154,40 | 1 |
| 163,20 | 2 | 164,07 | 2 |
| 178,61 | 3 | 179,56 | 3 |
| 193,37 | 4 | 190,31 | 4 |
| 199,95 | 5 | 199,86 | 5 |
| 207,32 | 6 | 209,33 | 6 |
| 218,29 | 7 | 217,82 | 7 |
| 226,43 | 8 | 226,87 | 8 |
| 235,46 | 9 | 236,06 | 9 |
| 243,40 | 10 | 241,41 | 10 |
| 253,50 | 11 | 252,25 | 11 |
| 263,20 | 12 | 261,65 | 12 |
| 268,10 | 13 | 275,32 | 13 |
| 282,01 | 14 | 283,47 | 14 |
| 299,96 | 15 | 292,35 | 15 |
| 309,38 | 16 | 305,50 | 16 |
| 321,90 | 17 | 315,46 | 17 |
| 334,61 | 18 | 327,36 | 18 |
| 354,52 | 19 | 346,80 | 19 |
| 368,60 | 20 | 362,10 | 20 |
| 383,04 | 21 | 376,04 | 21 |
| 412,68 | 22 | 392,55 | 22 |
| 452,60 | 23 | 418,64 | 23 |
| 491,78 | 24 | 450,49 | 24 |
| 536,58 | 25 | 487,70 | 25 |
| 590,74 | 26 | 540,75 | 26 |
| 647,48 | 27 | 594,65 | 27 |
| | | 675,80 | 28 |
| | | 765,67 | 29 |

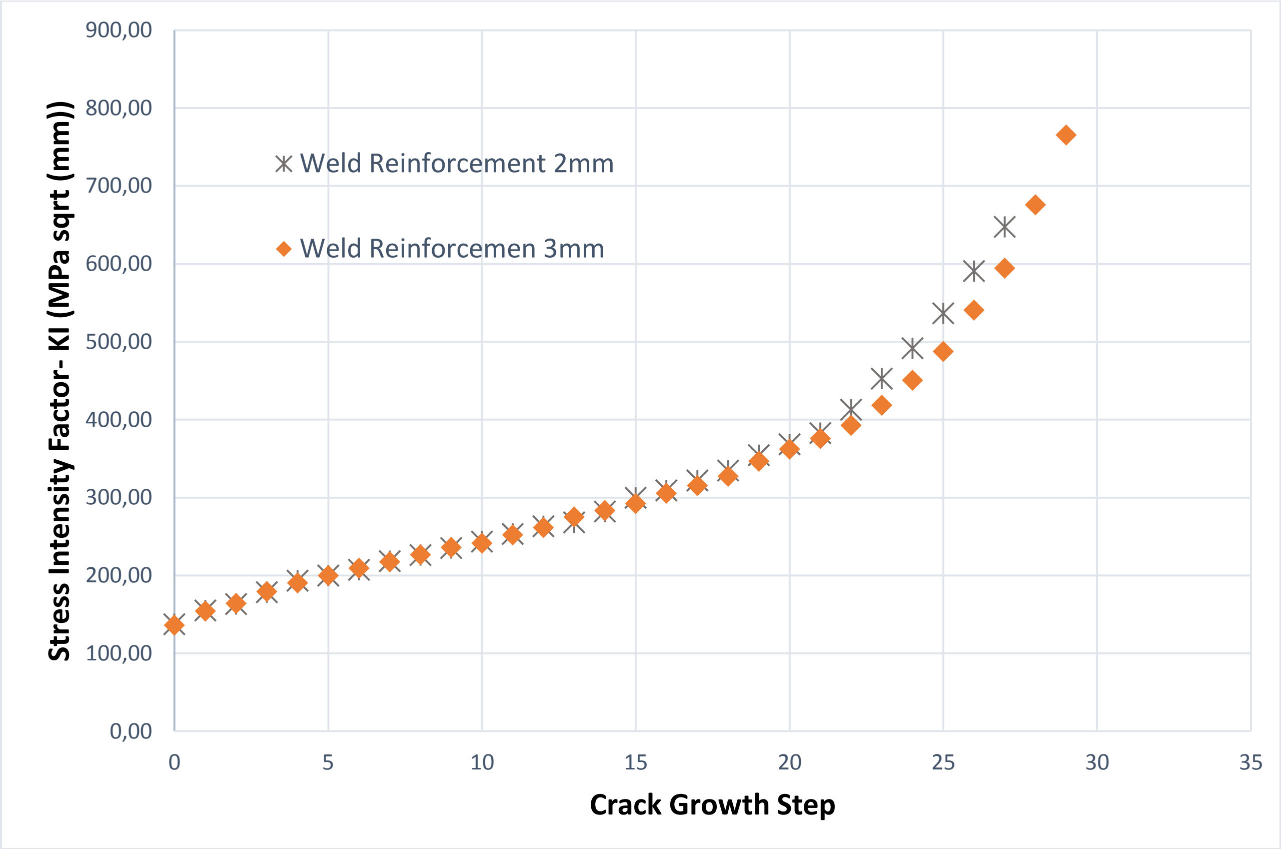

900,00
800,00
700,00
600,00
500,00
400,00
300,00
200,00
100,00
0,00
Stress Intensity Factor- KI (MPa sqrt (mm))
Weld Reinforcement 2mm
Weld Reinforcemen 3mm
0
5
10
15
20
25
30
35
Crack Growth Step
