# Supplementary material for: Computational modeling of fatigue crack propagation in butt welded joints subjected to axial load
Source: PLoS One. 2019 Jun 27;14(6):e0218973. doi: 10.1371/journal.pone.0218973 (PMC6597091; doi:10.1371/journal.pone.0218973)
Supplement: S4 Table — Paris Model parameters for crack propagation rate a) weld reinforcement 2mm, b) weld reinforcement 3mm. (PDF) [file pone.0218973.s004.pdf]

S4 Table. Paris Model parameters for Crack propagation rate a) weld reinforcement 2mm, b) weld reinforcement 3mm.

a) Weld reinforcement 2 mm

C	6,89E-12
n	3

Paris Law 2 mm

Length (mm)	KI (MPa sqrt(mm))
0	137,4733489
0,09541	154,9854733
0,22489	163,2015134
0,30673	178,6054106
0,38422	193,3731208
0,50865	199,9461995
0,62435	207,3212002
0,73153	218,2919004
0,84189	226,4268597
0,94424	235,4582298
1,0568	243,3994996
1,16827	253,496747
1,27759	263,2041805
1,42002	268,0956864

Step	da (m)	KIavg (MPa sqrt(m))	DK (MPa sqrt(m))	da/dN	DN	N
0	0	0	0	0,00E+00	0,00E+00	0,00E+00
1	0,00009541	4,624183422	4,161765079	5,63E-10	1,70E+05	1,70E+05
2	0,00012948	5,030981723	4,52788355	7,30E-10	1,77E+05	3,47E+05
3	0,00008184	5,404445999	4,864001399	9,11E-10	8,98E+04	4,37E+05
4	0,00007749	5,881501352	5,293351217	1,18E-09	6,55E+04	5,02E+05
5	0,00012443	6,218929102	5,597036191	1,41E-09	8,86E+04	5,91E+05
6	0,0001157	6,439467765	5,795520988	1,56E-09	7,39E+04	6,65E+05
7	0,00010718	6,729538979	6,056585081	1,79E-09	5,98E+04	7,25E+05
8	0,00011036	7,031626203	6,328463583	2,05E-09	5,38E+04	7,78E+05
9	0,00010235	7,303049904	6,572744913	2,31E-09	4,44E+04	8,23E+05
10	0,00011256	7,571411102	6,814269992	2,58E-09	4,36E+04	8,66E+05
11	0,00011147	7,856625313	7,070962782	2,89E-09	3,85E+04	9,05E+05
12	0,00010932	8,169765045	7,35278854	3,26E-09	3,35E+04	9,38E+05
13	0,00014243	8,400594716	7,560535244	3,56E-09	4,01E+04	9,78E+05

1,50025	282,0140088
1,55097	299,9568355
1,68644	309,3814349
1,79619	321,8990264
1,91052	334,6085998
1,96575	354,5237391
2,10688	368,5967285
2,24374	383,0429299
2,31797	412,6846344
2,58942	452,5966894
2,89497	491,7788275
3,08993	536,5809655
3,22074	590,7389233
3,4156	647,4755604

14	8,023E-05	8,698004436	7,828203992	3,96E-09	2,03E+04	9,99E+05
15	5,072E-05	9,201773809	8,281596428	4,71E-09	1,08E+04	1,01E+06
16	0,00013547	9,634491129	8,671042016	5,43E-09	2,50E+04	1,03E+06
17	0,00010975	9,981427885	8,983285097	6,06E-09	1,81E+04	1,05E+06
18	0,00011433	10,38030468	9,342274213	6,83E-09	1,67E+04	1,07E+06
19	5,523E-05	10,89614706	9,806532356	7,94E-09	6,96E+03	1,08E+06
20	0,00014113	11,43354696	10,29019226	9,21E-09	1,53E+04	1,09E+06
21	0,00013686	11,88447529	10,69602776	1,04E-08	1,32E+04	1,10E+06
22	7,423E-05	12,58156681	11,32341013	1,24E-08	6,00E+03	1,11E+06
23	0,00027145	13,68130912	12,31317821	1,60E-08	1,69E+04	1,13E+06
24	0,00030555	14,93189905	13,43870914	2,10E-08	1,45E+04	1,14E+06
25	0,00019496	16,25980803	14,63382723	2,73E-08	7,14E+03	1,15E+06
26	0,00013081	17,82450569	16,04205512	3,63E-08	3,61E+03	1,15E+06
27	0,00019486	19,57790449	17,62011404	4,85E-08	4,02E+03	1,16E+06

b) Weld reinforcement 3 mm

**C**	**6,89E-12**
**n**	**3**

Paris Law 3 mm

Length (mm)	KI (MPa sqrt(mm))
0	136,1875668
0,10685	154,3957086
0,23429	164,0749029
0,33135	179,5613039
0,44169	190,3125736
0,55268	199,8611343
0,66344	209,329879
0,77863	217,8205945
0,88588	226,8711913
0,99548	236,0574182
1,12171	241,4099842
1,21756	252,2537505
1,3239	261,6510594
1,39815	275,3210482

Step	da (m)	KIavg (MPa sqrt(m))	DK (MPa sqrt(m))	da/dN	DN	N
0	0	0	0	0,00E+00	0,00E+00	0,00E+00
1	0,00010685	4,5945284	4,13507556	4,87E-10	2,19E+05	2,19E+05
2	0,00012744	5,035466226	4,531919603	6,41E-10	1,99E+05	4,18E+05
3	0,00009706	5,43336952	4,890032568	8,06E-10	1,20E+05	5,39E+05
4	0,00011034	5,848223827	5,263401444	1,00E-09	1,10E+05	6,48E+05
5	0,00011099	6,169192565	5,552273308	1,18E-09	9,41E+04	7,42E+05
6	0,00011076	6,469882787	5,822894508	1,36E-09	8,14E+04	8,24E+05
7	0,00011519	6,753846997	6,078462298	1,55E-09	7,44E+04	8,98E+05
8	0,00010725	7,031199703	6,328079732	1,75E-09	6,14E+04	9,60E+05
9	0,0001096	7,319549416	6,587594474	1,97E-09	5,56E+04	1,02E+06
10	0,00012623	7,549428086	6,794485277	2,16E-09	5,84E+04	1,07E+06
11	0,00009585	7,805514775	7,024963298	2,39E-09	4,01E+04	1,11E+06
12	0,00010634	8,125554512	7,312999061	2,69E-09	3,95E+04	1,15E+06
13	7,425E-05	8,490280782	7,641252704	3,07E-09	2,42E+04	1,18E+06

1,5247	283,4706804
1,63975	292,3457392
1,75023	305,5028381
1,86654	315,4634435
1,98636	327,3613235
2,03614	346,8024057
2,16286	362,0971094
2,28373	376,0385797
2,4085	392,5535748
2,51471	418,642492
2,61712	450,4906441
2,97223	487,7014499
3,23713	540,7531165
3,47037	594,648289
3,57408	675,7961918
3,70048	765,6672374

14	0,00012655	8,835279537	7,951751584	3,46E-09	3,65E+04	1,21E+06
15	0,00011505	9,104463737	8,194017363	3,79E-09	3,04E+04	1,24E+06
16	0,00011048	9,452822994	8,507540695	4,24E-09	2,60E+04	1,27E+06
17	0,00011631	9,818346265	8,836511638	4,75E-09	2,45E+04	1,29E+06
18	0,00011982	10,16395952	9,147563568	5,27E-09	2,27E+04	1,32E+06
19	4,978E-05	10,65947239	9,593525148	6,08E-09	8,18E+03	1,33E+06
20	0,00012672	11,20869379	10,08782441	7,07E-09	1,79E+04	1,34E+06
21	0,00012087	11,67095864	10,50386277	7,98E-09	1,51E+04	1,36E+06
22	0,00012477	12,15251799	10,93726619	9,01E-09	1,38E+04	1,37E+06
23	0,00010621	12,82614549	11,54353094	1,06E-08	1,00E+04	1,38E+06
24	0,00010241	13,74221167	12,3679905	1,30E-08	7,86E+03	1,39E+06
25	0,00035511	14,83413048	13,35071743	1,64E-08	2,17E+04	1,41E+06
26	0,0002649	16,26130653	14,63517588	2,16E-08	1,23E+04	1,42E+06
27	0,00023324	17,95228578	16,1570572	2,91E-08	8,03E+03	1,43E+06
28	0,00010371	20,08750586	18,07875528	4,07E-08	2,55E+03	1,44E+06
29	0,0001264	22,79155486	20,51239938	5,95E-08	2,13E+03	1,44E+06
